# Association between pre-existing cardiometabolic comorbidities and the pathological profiles of breast cancer at initial diagnosis: a cross sectional study

**DOI:** 10.3332/ecancer.2022.1512

**Published:** 2023-02-23

**Authors:** Shridevi Subramaniam, Yek-Ching Kong, Cheng-Har Yip, Muthukkumaran Thiagarajan, Jayalakshmi Pailoor, Hafizah Zaharah, Nur Aishah Taib, Mee-Hoong See, Diana Sarfati, Nirmala Bhoo-Pathy

**Affiliations:** 1 Centre for Clinical Epidemiology, Institute for Clinical Research, National Institutes of Health, Ministry of Health, Shah Alam 40170, Malaysia; 2 Centre for Epidemiology and Evidence-Based Medicine, Department of Social and Preventive Medicine, Faculty of Medicine, Universiti Malaya, Kuala Lumpur 50603, Malaysia; 3 Subang Jaya Medical Centre, Subang Jaya 47500, Malaysia; 4 Department of Radiotherapy and Oncology, Kuala Lumpur Hospital, Ministry of Health, Kuala Lumpur 50586, Malaysia; 5 Department of Radiotherapy and Oncology, National Cancer Institute, Ministry of Health, Putrajaya 62250, Malaysia; 6 Department of Surgery, Faculty of Medicine, Universiti Malaya, Kuala Lumpur 50603, Malaysia; 7 National Director of Cancer Control, and Chief Executive Cancer Control Agency, PO Box 5013, Wellington 6140, New Zealand

**Keywords:** cancer, comorbidity, cardiometabolic, cardiovascular disease, pathology

## Abstract

The presence of comorbidities has been associated with later stages of breast cancer diagnosis. It is unclear whether biological mechanisms are partly responsible. We examined the association between the presence of pre-existing comorbidities and tumour profile at initial diagnosis with breast cancer. Data for the present analysis were derived from a prior inception cohort study comprising 2,501 multiethnic women, newly diagnosed with breast cancer between 2015 and 2017 in four hospitals across Klang Valley. At the inception of the cohort, medical and drug histories, height, weight and blood pressure were recorded. Blood samples were taken to measure serum lipid and glucose. Modified Charlson Comorbidity Index (CCI) was calculated using data extracted from medical records. The association of CCI as well as specific comorbidities, with pathological breast cancer profile was analysed. Higher comorbidity burden, namely cardiometabolic conditions were associated with unfavourable pathological features including larger tumours, involvement of >9 axillary lymph nodes, distant metastasis and human epidermal growth factor receptor 2 overexpression. These associations remained largely significant following multivariable analyses. Specifically, diabetes mellitus was independently associated with high nodal metastasis burden. Low level of high-density lipoprotein was associated with larger tumours (>5 cm), and distant metastasis. Evidence from this study seems to support the hypothesis that the later stages of breast cancer diagnosis in women with (cardiometabolic) comorbidities may be partially explained by underlying pathophysiological events.

## Introduction

Comorbidity is increasingly being recognised as an important prognostic factor following breast cancer, where it has been associated with adverse clinical outcomes and patient-centred outcomes [1–4]. Prior literature suggest that comorbidity may influence cancer stage at diagnosis both positively and negatively [5]. For instance, as cancer and common comorbidities share many similar risk factors and symptoms, any early cancer symptoms may be brushed off or misinterpreted as symptoms of the pre-existing health condition, leading to delayed cancer diagnosis and subsequently, more advanced cancer stages at diagnosis [6]. Conversely, frequent clinical encounters for management of comorbidity can also lead to earlier cancer diagnosis among those with pre-existing illness [7].

From a biological perspective, the presence of several overlapping pathophysiological mechanisms between common comorbidities and cancer may directly or indirectly impact stage at cancer diagnosis [8]. This is corroborated by evidence suggesting that the presence of comorbidity may result in local and systemic inflammation leading to alterations in the tumour microenvironment, which can exacerbate cell proliferation and carcinogenesis [9–11]. If biological mechanisms do indeed at least partly explain the association between comorbidities and stage at diagnosis in breast cancer, it is postulated that there will also be differences in distribution of its pathologic prognostic factors by comorbidity status. Additionally, such associations may be specific to certain types or categories of comorbid conditions such as the association between diabetes mellitus and cancer metastasis, which can be explained by the high circulating levels of insulin and insulin-like growth factors in people with diabetes [10].

Nonetheless, prior research examining the overlap between cancer and common comorbidities such as cardiovascular diseases appear to have largely focused on shared risk factors [12]. While most of the current evidence seems to imply that the association between comorbidity and cancer stage at diagnosis is largely explained by non-biological factors such as patient’s health behaviour, physician’s attitude and delivery of preventive care, it remains unclear whether the association may also be explained by biological factors. In this study, we had hypothesised that if biological mechanisms do indeed at least partly explain the association between comorbidities and stage at diagnosis in breast cancer, there will also be differences in the distribution of its pathologic prognostic factors by comorbidity status. We undertook a cross-sectional analysis of an inception cohort study to examine the association between pre-existing common comorbidities and tumour profile at initial breast cancer diagnosis. Specifically, we aimed to gain insights on whether there is evidence of unfavourable pathological features (tumour histology, pathological tumour size, nodal involvement, distant metastasis, tumour grade, hormone receptor status, human epidermal growth factor receptor 2 (HER2) status) among women with common comorbid conditions.

## Methods

### Study population

Data for the present analysis were derived from a prior inception cohort study, which recruited women newly diagnosed with breast cancer between 2015 and 2017 in four hospitals across Klang Valley, an urban conglomeration in Malaysia [13]. The sample for this study was drawn from various public and private hospitals in Malaysia; National Cancer Institute (national oncology referral centre), Kuala Lumpur Hospital (public general hospital), University Malaya Medical Centre (public university hospital) and Subang Jaya Medical Centre (private medical centre). This was expected to allow the recruitment of a diverse sample of Malaysians with breast cancer from various ethnic and socioeconomic backgrounds, which in turn are predictors of cardiovascular risk factors and other pre-existing comorbidities [14]. Patients with recurrent cancers were excluded.

### Study variables

Participants were recruited into the main study during their hospital visits. Demographic data including ethnicity (Malay, Chinese, Indian or other ethnicity), and age at diagnosis, as well as data on pre-existing medical conditions were collected via face-to-face interviews.

During the on-site screenings, height and body weight were measured. Body mass index (BMI) was calculated as weight divided by height squared (kg/m^2^). Obesity was recorded if the calculated BMI was 30 kg/m^2^ and above. Systolic and diastolic blood pressure (BP) readings were taken using an automated BP monitor. Hypertension was recorded based on patients’ self-report or if patients were on hypertensive medication and/or had a repeatedly elevated BP reading; systolic BP > 140 mmHg or diastolic BP > 90 mmHg (measured at least twice).

Non-fasting blood samples were drawn to measure lipid and glucose profiles. Sex specific cut-off of 1.3 mmol/L was used to define low high-density lipoprotein (HDL) as per clinical practice guidelines [15]. Diabetes was recorded based on self-report (verified with patient held-record), or if they were on diabetes medication and/or had a random blood glucose concentration of equal to or more than 11.0 mmol/L.

Date of cancer diagnosis was obtained from the medical records. Tumour profiles that were extracted from the pathological records included histology (invasive ductal carcinoma, invasive lobular carcinoma, others), pathological tumour size at presentation (≤5 cm, >5 cm, unknown) and number of positive axillary lymph nodes (pN: 0, 1–3, 4–9, ≥10, unknown). Tumour grading was determined using the Scarff–Bloom–Richardson classification (grade 1, 2 or 3, unknown). Presence of distant metastasis at initial diagnosis was determined radiologically (yes, no, unknown). Cancer stage included pathological/radiological Tumour, Node, Metastasis (TNM) staging based on American Joint Committee on Cancer (AJCC) 7th edition (I, II, III or IV). Hormonal receptor status was determined via immunohistochemistry and deemed positive when >1% of cells stained positive (progesterone and oestrogen receptor (ER) expression (positive, negative, unknown)). For HER2 status, only tumours with an immunohistochemistry score of 3+ were regarded as HER2 overexpressed (positive), whereas those with score of 1+ were deemed negative. Expressions of 2+ were regarded as equivocal and underwent further *in situ* hybridisation testing for HER2 gene amplification.

### Comorbidity measurement

In the current analysis, Charlson Comorbidity Index (CCI) was calculated for all patients using data on presence of comorbidities at baseline, which had been originally extracted from patients’ internal (hospital-based records) as well as external medical records (e.g. patient-held medical records, diabetes mini green book, discharge summaries) at the time of study recruitment, i.e. within 8 weeks of diagnosis [16]. While the original CCI is a weighted score that is based on both the number and severity of 19 pre-defined comorbid conditions including cancer, in the current study presence of cancer (weight = 1) or distant metastasis (weight = 6) was not scored. As a result, all patients were only scored if they had the following conditions: congestive heart failure (weight = 1), myocardial infarct (weight = 1), cerebrovascular disease (weight = 1), chronic pulmonary disease (weight = 1), paraplegia (weight = 2), dementia (weight = 1), diabetes without complications (weight = 1), diabetes with complications (weight = 2), mild liver disease (weight = 1), moderate or severe liver disease (weight = 3), peptic ulcer disease (weight = 1), peripheral vascular disease (weight = 1), rheumatologic disease (weight = 1), renal disease (weight = 2) and human immunodeficiency virus/acquired immune deficiency syndrome (weight = 6) [17].

Based on the presence of comorbidities, the corresponding weights were added to produce a cumulative score for every patient that we refer to as a modified CCI (ranging from 0 to 8). The score was stratified into quartiles with higher quartiles indicating higher comorbidity burden; Quartile 1 (score 0), Quartile 2 (score 1), Quartile 3 (score 3) and Quartile 4 (score ≥ 3).

In the current study, cardiometabolic comorbidities [18] included cardiovascular disease and diabetes mellitus [19] as well as their risk factors, namely hypertension, dyslipidaemia and obesity [20, 21].

### Statistical analysis

Descriptive statistics were used to summarise patient characteristics. The demographic and pathological profiles were compared across the patients based on their burden of comorbidities using ***χ***² test.

Multivariable logistic/linear regression analyses were used to determine the association between modified CCI quartiles and pathological risk factors. We also measured the association between specific comorbidities (hypertension, diabetes, obesity, low HDL level, pre-existing cardiovascular disease) and pathological characteristics.

Odds ratio (OR) with a 95% CI that does not include 1 was considered as statistically significant. Models were adjusted for age, ethnicity and type of hospital, as they may be associated with both comorbidity and pathological factors of breast cancer. Data were analysed using IBM Statistical Package for the Social Sciences version 26.

## Results

In the present analysis, 41 (1.6%) patients with incomplete information on pathological/radiological cancer staging were excluded, leaving 2,501 patients with invasive cancers (stage I–IV). Median age at diagnosis was 53 years (25th percentile: 45 years, 75th percentile: 62 years). A majority of patients were Chinese (54.8%), followed by Malays (32.0%), Indians (11.4%) and other ethnicities (1.8%). Median tumour size at presentation was 2.5 cm (25th percentile: 2.0 cm, 75th percentile: 4.0 cm), whereby a fifth of patients presented with tumours measuring larger than 5 cm. Approximately half of the patients had lymph node involvement at initial diagnosis. Overall, 24% presented with stage I disease at initial diagnosis, followed by 39% with stage II, 28% with stage III and 10% with stage IV breast cancer. Close to a quarter (24%) of the cohort comprised women presenting with late cancer stages (comprising stage IIIb, IIIc and IV).

Very few patients were classified as having a comorbidity based on self-report alone ((hypertension: 15), (hypercholesterolaemia: 9)). However, given that HDL level was measured solely from blood samples, data were missing in 20.6%.

About 65% of women with breast cancer were found to have at least one comorbidity at initial diagnoses, of which cardio-metabolic comorbidities were most prevalent. Common comorbidities comprised dyslipidaemia (67.5%), hypertension (44.3%) and diabetes (17.3%). About 18% were obese. Other comorbidities included obstructive airway diseases (*n* = 47), thyroid diseases (*n* = 32), peptic ulcer disease (*n* = 27), chronic kidney disease (*n* = 19) and liver diseases (*n* = 16).

Median score of the modified CCI was 1.0 (25th percentile: 0 score, 75th percentile: 2 score). Patients aged 65 or above were more likely to be in the higher quartiles of CCI (Table 1). Significant ethnic variations were observed where Indian women with breast cancer were more likely to present with higher prevalence of comorbidities compared to their Chinese and Malay counterparts (Table 1). Among the Indian patients with breast cancer, hypertension (56.7%), and diabetes mellitus (39.1%) were the most common comorbidities. Among the Malays, hypertension (53.1%) and obesity (32.2%) were most prevalent, whereas in the Chinese, hypertension (36.1%) and diabetes mellitus (8.9%) largely prevailed. Patients from public hospitals appeared to have more comorbidities compared to their counterparts managed in private hospital.

A higher proportion of patients with higher modified CCI scores presented with larger tumours compared to their counterparts with lower modified CCI scores (*p* = 0.002). Patients with lower modified CCI scores were more likely to present with pathologically favourable features compared to patients in the higher quartiles of modified CCI scores, such as progesterone receptor (PR) positive status and HER2 negative status. However, no associations were observed with tumour histology, tumour grade or ER status (Table 2). We also observed a significant association between higher quartiles of modified CCI and tumour size in linear regression analysis after adjusting for age; CCI quartile 4 versus CCI quartile 1: *B* = 0.91 (95% CI: 0.24, 1.58), *p* = 0.008 (results not shown). Likewise, higher quartiles of modified CCI were associated with increasing number of positive lymph nodes after adjustment for age, CCI quartile 3 versus CCI quartile 1: *B* = 1.01 (95% CI: 0.02, 2.01), *p* = 0.046 (results not shown). These associations nonetheless attenuated following additional adjustment for ethnicity and type of hospital.

Following adjustment for age, ethnicity and type of hospital, multivariable logistic regression analyses showed that those in the higher quartiles of modified CCI were more likely to present with high lymph node burden (>9 positive nodes) compared to quartile 1, with a corresponding adjusted OR of 1.51 (95% CI: 0.78–2.94) for quartile 2, 1.75 (95% CI: 0.83–3.69) for quartile 3 and 2.29 (95% CI: 1.01–5.18) for quartile 4; *p* for linear trend test = 0.064. While those in quartile 4 of the modified CCI also tended to be associated with larger tumours, higher odds of distant metastasis and late stages compared to those in the lowest quartile, findings were not statistically significant (Table 3). Tumour grade and HER2 overexpression however were not independently associated with modified CCI.

Several cardio-metabolic conditions were found to be specifically associated with unfavourable pathological profiles (Table 4). Low HDL levels, for instance, was associated with large tumours (>5 cm) (adjusted OR: 1.39, 95 CI%: 1.08–1.78), as well as distant metastasis (adjusted OR: 1.92, 95 CI%: 1.39–2.64), and hence late cancer stages at diagnoses (adjusted OR: 1.41, 95 CI%: 1.12–1.77). Likewise, hypercholesterolaemia was inversely associated with all the above factors. Diabetes mellitus was significantly associated with increased odds of higher nodal metastasis burden (pN3) at diagnosis (adjusted OR: 1.46, 95 CI%: 1.02–2.08). Although diabetes also appeared to be associated with distant metastasis, as well as late cancer stages at diagnoses, the findings were not statistically significant.

*Post hoc* analyses were also conducted where cardio-metabolic comorbidities, namely cardiovascular diseases, hypertension, diabetes, obesity and low HDL were grouped into three categories (0, 1, ≥2 comorbidities). Univariable logistic regression analysis showed that patients with no cardiometabolic comorbidities were significantly associated with favourable pathological features including smaller tumours (<2 cm), no nodal involvement and also absence of distance metastasis or HER2 overexpression, compared to their counterparts with two or more (clustering) cardiometabolic comorbidities (results not shown). Multivariable analyses revealed that compared to breast cancer patients with neither cardiovascular diseases, hypertension, diabetes, low HDL nor obesity at baseline, women who presented with clustering of these comorbid conditions were more likely to be associated with larger tumours, distant metastasis and late cancer stages (Table 5).

Sensitivity analyses adjusting for treatment with antihypertensives, statin and antidiabetic drugs in the multivariable analyses examining the association of hypertension, hypercholesterolaemia/low HDL and diabetes with distant metastasis/cancer stage, respectively, did not materially change the study inferences.

## Discussion

In this relatively young cohort of women who were newly diagnosed with breast cancer, close to two-thirds were found to have at least one comorbidity at initial presentation. Apart from age, ethnicity was also significantly associated with baseline burden of comorbidities. The presence of cardio-metabolic comorbidities seemed to be independently associated with unfavourable pathological features at initial breast cancer diagnosis including larger tumours, higher lymph node involvement and distant metastasis. Specifically, low levels of HDL were significantly associated with larger tumours, distant metastasis and late cancer stages, while presence of diabetes mellitus at baseline was significantly associated with higher nodal metastasis burden.

Our findings of higher prevalence of cardiometabolic comorbidities among women with breast cancer who are of Indian ethnicity tally with the reports from the recent National Health and Morbidity Survey, which is a nationwide study of the general Malaysian population [22]. The ethnic differences, apart from being attributed to variations in dietary preferences and lifestyle behaviours [23], may also be explained by genetic factors. For instance, genetic variants that are associated with increased risks of cardiovascular diseases have been found to be more prevalent among people of Indian origin [24]. It is thought that all of the above may also apply to Indian women with breast cancer.

While the association between comorbidity and cancer stage at diagnosis is well-established, the direction and magnitude of the association appear to vary [3]. Comorbidities may influence tumour biology through direct and indirect mechanisms, including manifestation of local and systemic inflammation, and alterations to tumour microenvironment [9]. Conceivably, these pathophysiological events may in turn influence carcinogenesis and progression of cancer including its propensity to metastasise. This is well supported by our present findings where positive associations were observed between higher comorbidity burden and more aggressive pathological features including, higher nodal burden and presence of distant metastasis. The associations were less apparent when all comorbidities were included for distant metastasis comparing quartile 4 of modified CCI versus quartile 1, indicating that there may be specific biological mechanisms that apply only for a specific subset of comorbidities. This is further supported by our *post hoc* analyses including only the cardiovascular diseases, hypertension, diabetes, obesity and low HDL, where we have demonstrated significantly stronger findings for distant metastasis comparing those with ≥2 cardiometabolic diseases versus no cardiometabolic disease. While it is acknowledged that certain medications to treat common comorbidities, such as statins and anti-inflammatory agents may confer a protective effect against cancer progression [25–27], sensitivity analyses adjusting for use of such medications did not appear to alter study inferences.

The significant association between low HDL and risk of distant metastasis that we have observed appears to be in line with prior literature, namely from large clinical trials where an inverse association between HDL levels and risk of breast cancer has been reported [11]. In a study comparing women with breast cancer and healthy controls, significantly lower levels of HDL and elevated levels of total cholesterol were reported in those with breast cancer [28]. It has been posited that lower levels of HDL influence carcinogenesis via cell entry cycle regulation, cytokine production and antioxidation [29]. *In vitro* studies have also reported that under oxidative stress, oxidatively modified HDL can promote cell proliferation, migration, invasion and adhesion, all of which may contribute to tumour progression [11].

Multiple mechanisms have been proposed in explaining how diabetes could influence cancer progression, including hyperinsulinaemia, hyperglycaemia and chronic inflammation [10]. The activation of high circulating levels of insulin and insulin-like growth factors among people with diabetes, both of which promote cell proliferation and affect cell apoptosis, has been linked with a higher risk of incident cancer, as well as cancer metastasis and recurrence [10]. Furthermore, it has been shown that the composition and structure of HDL are often altered in patients with diabetes, leading to increased proinflammatory actions [11].

While it appeared that cancer stage at diagnosis was not significantly different between the various quartiles of CCI, more meaningful differences were observed when analyses were confined to cardio-metabolic comorbidities. Moreover, associations were even more apparent when specific comorbidities were examined in relation to specific tumour characteristics (tumour size, nodal involvement, distant metastasis). It must be further noted that certain conditions that can only be measured via laboratory tests were also found to be associated with aggressive tumour features. Intuitively, the bigger the tumour, the easier it should be for women to self-detect it. However, as we did not have data on breast cancer screening behaviour, we were unable to determine the relative role and magnitude of biological and non-biological factors in influencing cancer stage at diagnosis. While it is often not straightforward to disentangle the interplay between biological and non-biological factors in influencing cancer stage at diagnosis, our present findings at the least challenge the notion that the association between cardio-metabolic comorbidities and advanced cancer stages at diagnoses is explained purely by delays in cancer diagnosis.

### Strengths and limitations

To the best of authors’ knowledge, this is the first study that consecutively measured the baseline prevalence of common comorbidities in women who were newly diagnosed with breast cancer, which were then examined in relation to their clinicopathological characteristics at initial diagnosis. Nonetheless, it is acknowledged that there are intersections between many comorbid conditions, and it is plausible that some of the associations are bidirectional, rendering it difficult to disentangle their effects. Furthermore, the CCI is limited in terms of the comorbid conditions that it encompasses. Despite its shortcoming, the CCI is still commonly used due to a lack of a gold standard for measuring comorbidities in cancer populations [30]. Last but not least, it remains possible that we may have missed some comorbidities, particularly early disease or less severe conditions which may have been undiagnosed.

## Conclusion

Taken together, our study findings seem to point towards the presence of underlying biological mechanisms linking cardiometabolic comorbidities and pathological profiles of breast cancer among women who are newly diagnosed. This in turn implies that the late stage at cancer diagnosis that is observed in women with breast cancer may not only be attributed to delay in timeliness of diagnoses arising from patient, physician and health systems-related factors [4], but also by pathophysiological interactions between common comorbidities such as dyslipidaemia as well as diabetes, and tumour biology. Given the poorer survival among breast cancer patients with comorbidities, there is a need for more evidence to understand the interactions, magnitude and impact of both socio-structural and biological factors that lead to more aggressive cancers at diagnosis [1, 2]. From a healthcare system’s point of view, there needs to be a paradigm shift from focusing on cancer patients with a narrow lens (single disease approach) to a wider lens that encompasses the management of multimorbidities. A coordinated care model therefore will lead to timely diagnosis and optimal management of morbidities, which in turn will result in improved clinical and patient-centred outcomes, as well as a reduction in healthcare costs for both patients and healthcare systems [31, 32].

## Authors’ contributions

NB: Concept and design, statistical analysis, interpretation of data, drafting of manuscript, critical revision of manuscript and final approval. SS: Data acquisition, statistical analysis, interpretation of data, drafting of manuscript, critical revision of manuscript and final approval. YCK: Data acquisition, drafting of manuscript, critical revision of manuscript and final approval. CHY: Data acquisition, interpretation of data, critical revision of manuscript and final approval. MT: Critical revision of manuscript and final approval. PJ: Critical revision of manuscript and final approval. HZ: Data acquisition, critical revision of manuscript and final approval. NAT: Data acquisition, critical revision of manuscript and final approval. MHS: Data acquisition, critical revision of manuscript and final approval. DS: Statistical analysis, interpretation of data, drafting of manuscript, critical revision of manuscript and final approval.

## Conflicts of interest

The authors have no conflicts of interest to report.

## Funding

The authors have no funding to declare for this work.

## Ethics approval

Ethical approval was approved by the Medical Research and Ethics Committee (NMRR-15-803-24756) and University of Malaya Research Ethics Committee (UMMEC 896.150). Written informed consent was obtained from all participants. Ethical principles according to Helsinki’s declaration were observed throughout the conduct of the study.

## Figures and Tables

**Table 1.** Baseline characteristics of women newly diagnosed with breast cancer by comorbidity burdena.

Sociodemographic factors	Overall (*N* = 2,501)		Modified CCI^a^								*p*-value^b^
			Quartile 1		Quartile 2		Quartile 3		Quartile 4		
	*n*	%	*n*	%	*n*	%	*n*	%	*n*	%	
Age at diagnosis (years)											
<40	269	10.8	258	29.4	7	1.0	3	0.6	1	0.2	<0.001
40–49	686	27.4	619	70.4	60	9.2	5	0.9	2	0.5	
50–64	1,096	43.8	2	0.2	587	89.8	369	69.6	138	31.5	
≥65	450	18.0	0	0.0	0	0.0	153	28.9	297	67.8	
Ethnicity											
Malay	803	32.0	306	34.8	205	31.3	166	31.3	126	28.8	<0.001
Chinese	1,370	54.8	487	55.5	384	58.7	295	55.7	204	46.6	
Indian	284	11.4	69	7.8	56	8.6	64	12.1	95	21.7	
Others	44	1.8	17	1.9	9	1.4	5	0.9	13	3.0	
Hospital											
Hospital Kuala Lumpur (public hospital)	739	29.5	200	22.8	182	27.8	179	33.8	178	40.6	<0.001
University Malaya Medical Centre (public university hospital)	345	13.8	90	10.2	78	12.0	74	14.0	103	23.5	
National Cancer Institute (public hospital)	267	10.7	98	11.1	63	09.6	63	11.9	43	9.9	
Subang Jaya Medical Centre (private hospital)	1,150	46.0	491	55.9	331	50.6	214	40.3	114	26.0	

a Comorbidity burden is expressed by quartiles of modified CCI, in which patients were not assigned weights for having cancer (score of 2), or metastatic disease (score of 6) but only assigned scores if they had concurrent illnesses. Quartile 1 (score ≤ 0), Quartile 2 (score 1), Quartile 3 (score 2) and Quartile 4 (score ≥ 3). Higher quartiles indicate higher comorbidity burden

b Derived using chi square test: p-value < 0.05 was considered as statistically significant

**Table 2.** Clinicopathological features of breast cancer by comorbidity burden in 2,501 women.

Breast cancer profile at initial diagnosis	Total		Modified CCI ^a^								*p*-value^b^
			Quartile 1		Quartile 2		Quartile 3		Quartile 4		
	*n*	%	*n*	%	*n*	%	*n*	%	*n*	%	
Histology											
Invasive ductal carcinoma	2,263	90.5	797	90.7	597	91.3	474	89.4	395	90.2	0.329
Invasive lobular carcinoma	97	3.9	25	2.8	26	4.0	27	5.1	19	4.3	
Others	141	5.6	57	6.5	31	4.7	29	5.5	24	5.5	
Tumour size											
<2 cm	566	23.4	222	25.9	158	25.0	116	22.6	70	16.8	0.002
2–5 cm	1,427	58.9	476	55.5	357	56.4	312	60.8	282	67.6	
>5 cm	428	17.7	160	18.6	118	18.6	85	16.6	65	15.6	
Lymph node involvement											
None	1,255	50.7	451	51.8	319	49.5	260	49.6	225	51.5	0.610
1–3 nodes	661	26.7	229	26.3	184	28.5	146	27.9	102	23.3	
4–9 nodes	311	12.6	105	12.1	74	11.5	65	12.4	67	15.3	
≥10 nodes	249	10.0	85	9.8	68	10.5	53	10.1	43	9.8	
Distant metastasis											
Yes	251	10.0	75	8.5	64	9.8	62	11.7	50	11.4	0.188
No	2,250	90.0	804	91.5	590	90.2	468	88.3	388	88.6	
TNM staging											
Stage 1	600	24.0	230	26.2	165	25.2	127	24.0	78	17.8	0.059
Stage 2	964	38.5	339	38.6	243	37.2	195	36.8	187	42.7	
Stage 3	686	27.5	235	26.7	182	27.8	146	27.5	123	28.1	
Stage 4	251	10.0	75	8.5	64	9.8	62	11.7	50	11.4	
Tumour grade											
1 (low)	233	10.4	72	9.1	57	9.9	52	10.7	52	13.5	0.076
2 (intermediate)	1,146	51.2	395	49.9	291	50.3	252	52.1	208	54.0	
3 (high)	859	38.4	324	41.0	230	39.8	180	37.2	125	32.5	
ER status											
Positive	1,706	70.3	605	71.2	414	65.9	366	70.9	321	74.3	0.023
Negative	720	29.7	245	28.8	214	34.1	150	29.1	111	25.7	
PR status											
Positive	1,353	55.8	518	61.0	307	48.8	278	53.9	250	58.1	<0.001
Negative	1,071	44.2	331	39.0	322	51.2	238	46.1	180	41.9	
HER2											
Positive	784	33.3	245	29.3	226	36.9	165	33.1	148	36.4	0.010
Negative	1,570	66.7	591	70.7	387	63.1	333	66.9	259	63.6	

a Comorbidity burden is expressed by quartiles of modified CCI, in which patients were not assigned weights for having cancer (score of 2), or metastatic disease (score of 6) but only assigned scores if they had concurrent illnesses. Quartile 1 (score 0), Quartile 2 (score 1), Quartile 3 (score 2) and Quartile 4(score ≥ 3). Higher quartiles indicate higher comorbidity burden

b Derived using chi square test

**Table 3.** Association between baseline comorbidity burden^a^ and pathological profile of breast cancer.

Comorbidities at baseline	Tumour >5 cm (*n* = 2,421)		Adjusted OR^b^ (95% CI)	*p*-value^c^	*p* for trend^d^
	No (%)	Yes (%)			
Modified CCI quartile 1	698 (35.0)	160 (37.4)	Ref	Ref	0.527
Modified CCI quartile 2	515 (25.8)	0118 (27.6)	1.63 (0.94–2.82)	0.082	
Modified CCI quartile 3	428 (21.5)	085 (19.9)	1.38 (0.74–2.60)	0.315	
Modified CCI quartile 4	352 (17.7)	065 (15.1)	1.22 (0.61–2.43)	0.582	
	**Axillary lymph node involvement > 9 (*n* = 2,476)**		**Adjusted OR^e^ (95% CI)**	***p*-value^c^**	***p* for trend^d^**
	**No (%)**	**Yes (%)**			
Modified CCI quartile 1	788 (35.2)	85 (34.1)	Ref	Ref	0.064
Modified CCI quartile 2	577 (25.9)	68 (27.3)	1.51 (0.78–2.94)	0.184	
Modified CCI quartile 3	471 (21.1)	53 (21.3)	1.75 (0.83–3.69)	0.123	
Modified CCI quartile 4	394 (17.7)	043 (17.3)	2.29 (1.01–5.18)	0.046	
	**Distant metastasis (*n* = 2,501)**		**Adjusted OR^f^ (95% CI)**	***p*-value^c^**	***p* for trend^d^**
	**No (%)**	**Yes (%)**			
Modified CCI quartile 1	804 (35.7)	75 (29.9)	Ref	Ref	0.370
Modified CCI quartile 2	590 (26.2)	64 (25.5)	1.37 (0.67–2.77)	0.395	
Modified CCI quartile 3	468 (20.8)	62 (24.7)	1.65 (0.76–3.62)	0.219	
Modified CCI quartile 4	388 (17.2)	50 (19.9)	1.56 (0.67–3.65)	0.304	
	**Cancer stage^g^(*n* =2,501)**		**Adjusted OR ^h^ (95% CI)**	***p*-value^c^**	***p* for trend^d^**
	**Early (%)**	**Late (%)**			
Modified CCI quartile 1	701 (36.6)	178 (30.4)	Ref	Ref	0.080
Modified CCI quartile 2	498 (26.0)	156 (26.7)	1.45 (0.86–2.44)	0.161	
Modified CCI quartile 3	393 (20.5)	137 (23.4)	1.68 (0.95–2.99)	0.077	
Modified CCI quartile 4	324 (16.9)	114 (19.5)	1.84 (0.99–3.41)	0.055	
	**High grade tumour (*n* = 2,238)**		**Adjusted OR ^i^ (95% CI)**	***p*-value^c^**	***p* for trend^d^**
	**No (%)**	**Yes (%)**			
Modified CCI quartile 1	467 (33.9)	324 (37.7)	Ref	Ref	0.490
Modified CCI quartile 2	348 (25.2)	230 (26.8)	0.72 (0.42–1.23)	0.230	
Modified CCI quartile 3	304 (22.0)	180 (21.0)	0.70 (0.39–1.27)	0.244	
Modified CCI quartile 4	260 (18.9)	125 (14.5)	0.68 (0.36–1.29)	0.238	
	**PR positive (n = 2,424)**		**Adjusted OR ^j^ (95% CI)**	***p*-value^c^**	**p for trend^d^**
	**No (%)**	**Yes (%)**			
Modified CCI quartile 1	331 (30.9)	518 (38.3)	Ref	Ref	0.029
Modified CCI quartile 2	322 (30.1)	307 (22.7)	1.00 (0.61–1.64)	0.995	
Modified CCI quartile 3	238 (22.2)	278 (20.5)	1.26 (0.73–2.17)	0.409	
Modified CCI quartile 4	180 (16.8)	250 (18.5)	1.44 (0.80–2.60)	0.222	
	**HER2 overexpression^k^** **(*n* = 2,354)**		**Adjusted OR^l^ (95% CI)**	***p*-value^c^**	***p* for trend^d^**
	**No (%)**	**Yes (%)**			
Modified CCI quartile 1	591 (37.6)	245 (31.2)	Ref	Ref	0.220
Modified CCI quartile 2	387 (24.6)	226 (28.8)	1.19 (0.70–2.02)	0.515	
Modified CCI quartile 3	333 (21.3)	165 (21.0)	0.92 (0.51–1.64)	0.771	
Modified CCI quartile 4	259 (16.5)	148 (19.0)	0.93 (0.50–1.75)	0.830	

a Comorbidity burden is expressed by quartiles of modified CCI, in which patients were not assigned weights for having cancer (score of 2), or metastatic disease (score of 6) but only assigned scores if they had concurrent illnesses

b Derived using multivariable logistic regression analysis with pathological tumour size > 5 cm as the outcome, adjusted for age (categorical), ethnicity and type of hospital

c Derived using multivariable logistic regression, p value < 0.05 was considered as statistically significant

d p for trend was computed by entering the quartiles as a continuous term (1, 2, 3, 4) in the logistic regression model

e Derived using multivariable logistic regression analysis with axillary nodal involvement of 10 or more as the outcome, adjusted for age (categorical), ethnicity and type of hospital

f Derived using multivariable logistic regression analysis with presence of distant metastasis as the outcome, adjusted for age (categorical), ethnicity and type of hospital

g Early stage includes TNM stage I, II and IIIa, whereas late stage includes TNM stage IIIb, IIIc and IV

h Derived using multivariable logistic regression analysis with late stage as the outcome, adjusted for age (categorical), ethnicity and type of hospital

i Derived using multivariable logistic regression analysis with presence of high-grade tumour as the outcome, adjusted for age (categorical), ethnicity and type of hospital

j Derived using multivariable logistic regression analysis with presence of PR positive as the outcome, adjusted for age (categorical), ethnicity and type of hospital

k HER2, Human epidermal growth factor receptor 2

l Derived using multivariable logistic regression analysis with presence of HER2 overexpression as the outcome, adjusted for age (categorical), ethnicity and type of hospital

**Table 4.** Association between baseline cardio-metabolic comorbidities and the tumour profile at initial diagnosis with breast cancer.

Cardiovascular comorbidities	Tumour >5 cm (*n* = 2,421)		*p*-value^a^	Adjusted OR^b^ (95% CI)
	No	Yes		
	*n* = 1993	*n* = 428		
Hypertension				
No	1,102 (55.4)	250 (58.8)	Ref	Ref
Yes	886 (44.6)	175 (41.2)	0.100	0.82 (0.64–1.04)
Diabetes mellitus				
No	1,659 (83.3)	352 (82.2)	Ref	Ref
Yes	332 (16.7)	76 (17.8)	0.972	1.01 (0.74–1.36)
Obesity				
No	1,573 (82.2)	324 (80.4)	Ref	Ref
Yes	341 (17.8)	79 (19.6)	0.739	0.95 (0.71–1.27)
Low high-density lipoprotein				
No	1,061 (66.1)	185 (56.4)	Ref	Ref
Yes	545 (33.9)	143 (43.6)	0.010	1.39 (1.08–1.78)
Hypercholesterolaemia				
No	923 (48.1)	215 (52.6)	Ref	Ref
Yes	995 (51.9)	194 (47.4)	0.836	0.84 (0.67–1.05)
Cardiovascular disease				
No	1,917 (96.2)	407 (95.1)	Ref	Ref
Yes	76 ( 3.8)	21(4.9)	0.348	1.28 (0.77–2.13)
	**Axillary lymph node involvement > 9 (N3) (*n* = 2,476)**		***p*-value^a^**	**Adjusted OR^c^ (95% CI)**
	**No**	**Yes**		
	***n* = 2,227**	***n* = 249**		
Hypertension				
No	1,245 (56.0)	130 (52.8)	Ref	Ref
Yes	977 (44.0)	116 (47.2)	0.427	1.13 (0.84–1.53)
Diabetes mellitus				
No	1,855 (82.3)	192 (77.4)	Ref	Ref
Yes	371 (16.7)	56 (22.6)	0.037	1.46 (1.02–2.08)
Obesity				
No	1,754 (82.3)	189 (79.1)	Ref	Ref
Yes	377 (17.7)	50 (20.9)	0.969	1.01 (0.71–1.43)
Low high-density lipoprotein				
No	1,135 (63.8)	127 (66.5)	Ref	Ref
Yes	645 (36.2)	64 (33.5)	0.361	0.86 (0.62–1.19)
Hypercholesterolaemia				
No	1,048 (48.9)	123 (51.2)	Ref	Ref
Yes	1,096 (51.1)	117 (48.8)	0.284	0.86 (0.65–1.13)
Cardiovascular disease				
No	2,134 (95.8)	239 (96.0)	Ref	Ref
Yes	93 (4.2)	10 (4.0)	0.897	0.96 (0.48–1.90)
**Cardiovascular comorbidities**	**Distant metastasis (*n* = 2,501)**		***p*-value^a^**	**Adjusted OR^d^ (95% CI)**
	**No**	**Yes**		
	***n* = 2,250**	***n* = 251**		
Hypertension				
No	1,261 (56.2)	128 (51.2)	Ref	Ref
Yes	982 (43.8)	122 (48.8)	0.782	0.96 (0.71–1.29)
Diabetes mellitus				
No	1,871 (83.2)	196 (78.1)	Ref	Ref
Yes	377 (16.8)	55 (21.9)	0.614	1.10 (0.77–1.56)
Obesity				
No	1,770 (81.8)	188 (82.5)	Ref	Ref
Yes	393 (18.2)	40 (17.5)	0.126	0.75 (0.51–1.09)
Low high-density lipoprotein				
No	1,182 (65.4)	84 (46.9)	Ref	Ref
Yes	624 (34.6)	95 (53.1)	<0.001	1.92 (1.39–2.64)
Hypercholesterolaemia				
No	1,051 (48.5)	136 (56.4)	Ref	Ref
Yes	1,115 (51.5)	104 (43.6)	0.006	0.67 (0.51–0.89)
Cardiovascular disease				
No	2,164 (96.2)	233 (92.8)	Ref	Ref
Yes	86 (3.8)	18 (7.2)	0.096	1.59 (0.92–2.73)
	**Cancer stage^e^ (*n* = 2,501)**		***p*-value^a^**	**Adjusted OR^f^ (95% CI)**
	**Early**	**Late**		
	***n* = 1,916**	***n* = 585**		
Hypertension				
No	1,087 (56.9)	302 (52.0)	Ref	Ref
Yes	825 (43.1)	279 (48.0)	0.898	0.99 (0.80–1.22)
Diabetes mellitus				
No	1,610 (84.1)	457 (78.3)	Ref	Ref
Yes	305 (15.9)	127 (21.7)	0.163	1.20 (0.93–1.55)
Obesity				
No	1,512 (82.0)	446 (81.5)	Ref	Ref
Yes	332 (18.0)	101 (18.5)	0.086	0.80 (0.61–1.03)
Low high-density lipoprotein				
No	1,027 (66.0)	239 (55.8)	Ref	Ref
Yes	530 (34.0)	189 (44.2)	0.003	1.41 (1.12–1.77)
Hypercholesterolaemia				
No	895 (48.5)	292 (51.9)	Ref	Ref
Yes	949 (51.5)	271 (48.1)	0.031	0.80 (0.66–0.98)
Cardiovascular disease				
No	1,837 (95.9)	560 (95.7)	Ref	Ref
Yes	79 (4.1)	25 (4.3)	0.480	0.84 (0.53–1.35)

a Derived using multivariable logistic regression, p value < 0.05 was considered as statistically significant

b Derived using multivariable logistic regression analysis with pathological tumour size >5 cm as the outcome, adjusted for age (categorical), ethnicity and type of hospital

c Derived using multivariable logistic regression analysis with axillary nodal involvement of 10 or more as the outcome, adjusted for age (categorical), ethnicity and type of hospital

d Derived using multivariable logistic regression analysis with distant metastasis as the outcome, adjusted for age (categorical), ethnicity and type of hospital

e Early stage includes TNM stage I, II and IIIa, whereas late stage includes TNM stage IIIb, IIIc and IV

f Derived using multivariable logistic regression analysis with late stage as the outcome, adjusted for age (categorical), ethnicity and type of hospital

**Table 5.** Association between clustering of cardio-metabolic comorbidities at baseline and the tumour profile at initial diagnosis with breast cancer.

Number of cardio-metabolic risk factors^a^	Tumour ≥5 cm		Adjusted OR^b^(95% CI)	*p*-value^c^	*p* for trend^d^
	No(%)	Yes(%)			
0	571 (36.5)	94 (29.8)	Ref	Ref	0.093
1	487 (31.1)	0100 (31.7)	1.22 (0.89–1.68)	0.212	
≥2	507 (32.4)	0121 (38.4)	1.33 (0.95–1.87)	0.093	
	**Axillary lymph node involvement > 9 (pN3)**		**Adjusted OR^e^(95% CI)**	***p*-value^c^**	***p* for trend^d^**
	**No(%)**	**Yes(%)**			
0	616 (35.6)	57 (30.6)	Ref	Ref	0.401
1	532 (30.8)	64 (34.4)	1.34 (0.91–1.97)	0.137	
≥2	580 (33.6)	65 (34.9)	1.19 (0.78–1.82)	0.413	
	**Distant metastasis**		**Adjusted OR^f^(95% CI)**	***p*-value^c^**	***p* for trend^d^**
	**No(%)**	**Yes(%)**			
0	630 (35.8)	44 (25.9)	Ref	Ref	0.017
1	558 (31.7)	42 (24.7)	0.99 (0.63–1.55)	0.954	
≥2	570 (32.5)	84 (49.4)	1.65 (1.07–2.54)	0.024	
	**Cancer stage^g^**		**Adjusted OR^h^(95% CI)**	***p*-value^c^**	***p* for trend^d^**
	**Early*n* (%)**	**Late*n* (%)**			
0	561 (37.0)	113 (27.4)	Ref	Ref	0.057
1	468 (30.9)	132 (32.0)	1.29 (0.96–1.72)	0.087	
≥2	486 (32.1)	168 (40.7)	1.35 (0.99–1.83)	0.054	
	**HER2 overexpression^i^**		**Adjusted OR^j^(95% CI)**	***p*-value^c^**	***p* for trend^d^**
	**No*n* (%)**	**Yes*n* (%)**			
0	473 (37.3)	183 (32.0)	Ref	Ref	0.547
1	411 (32.4)	161 (28.1)	0.85 (0.65–1.11)	0.237	
≥2	383 (30.3)	228 (39.9)	0.93 (0.70–1.21)	0.547	

a Include cardiovascular disease, hypertension, diabetes, obesity and low level of HDL

b Derived using multivariable logistic regression analysis with pathological tumour size > 5 cm as the outcome, adjusted for age (categorical), ethnicity and type of hospital

c Derived using multivariable logistic regression, *p* value < 0.05 was considered as statistically significant

d *p* for trend was computed by entering the number of cardio-metabolic comorbidities as a continuous term in the logistic regression model

e Derived using multivariable logistic regression analysis with axillary nodal involvement of 10 or more as the outcome, adjusted for age (categorical), ethnicity and type of hospital

f Derived using multivariable logistic regression analysis with presence of distant metastasis as the outcome, adjusted for age (categorical), ethnicity and type of hospital

g Early stage includes TNM stage 1, 2 and 3a, whereas late stage includes TNM stage 3b, 3c and 4

h Derived using multivariable logistic regression analysis with late stage as the outcome, adjusted for age (categorical), ethnicity and type of hospital

i HER2, Human epidermal growth factor receptor 2

j Derived using multivariable logistic regression analysis with presence of HER2 overexpression as the outcome, adjusted for age (categorical), ethnicity and type of hospital

## References

[1] Braithwaite D, Moore DH, Satariano WA (2012). Prognostic impact of comorbidity among long-term breast cancer survivors: results from the LACE study. Cancer Epidemiol Biomarkers Prev.

[2] Land LH, Dalton SO, Jørgensen TL (2012). Comorbidity and survival after early breast cancer. A review. Crit Rev Oncol Hematol.

[3] Sarfati D, Koczwara B, Jackson C (2016). The impact of comorbidity on cancer and its treatment. CA Cancer J Clin.

[4] Lee L, Cheung WY, Atkinson E (2011). Impact of comorbidity on chemotherapy use and outcomes in solid tumors: a systematic review. J Clin Oncol.

[5] Renzi C, Kaushal A, Emery J (2019). Comorbid chronic diseases and cancer diagnosis: disease-specific effects and underlying mechanisms. Nat Rev Clin Oncol.

[6] Gurney J, Sarfati D, Stanley J (2015). The impact of patient comorbidity on cancer stage at diagnosis. Br J Cancer.

[7] Vaeth PA, Satariano WA, Ragland DR (2000). Limiting comorbid conditions and breast cancer stage at diagnosis. J Gerontol A Biol Sci Med Sci.

[8] Fleming ST, Pursley HG, Newman B (2005). Comorbidity as a predictor of stage of illness for patients with breast cancer. Med Care.

[9] Panigrahi G, Ambs S (2021). How comorbidities shape cancer biology and survival. Trends Cancer.

[10] Giovannucci E, Harlan DM, Archer MC (2010). Diabetes and cancer: a consensus report. Diabetes Care.

[11] Cedó L, Reddy ST, Mato E (2019). HDL and LDL: potential new players in breast cancer development. J Clin Med.

[12] Johnson CB, Davis MK, Law A (2016). Shared risk factors for cardiovascular disease and cancer: implications for preventive health and clinical care in oncology patients. Can J Cadiol.

[13] Subramaniam S, Kong YC, Zaharah H (2021). Baseline cardiovascular comorbidities, and the influence on cancer treatment decision-making in women with breast cancer. Ecancermedicalscience.

[14] Su TT, Amiri M, Mohd Hairi F (2015). Prediction of cardiovascular disease risk among low-income urban dwellers in metropolitan Kuala Lumpur, Malaysia. Biomed Res Int.

[15] Ministry of Health Malaysia (2017). Clinical Practice Guidelines. Management of Dyslipidaemia.

[16] Ramli AS, Selvarajah S, Daud MH (2016). Effectiveness of the EMPOWER-PAR intervention in improving clinical outcomes of type 2 diabetes mellitus in primary care: a pragmatic cluster randomised controlled trial. BMC Fam Pract.

[17] Charlson ME, Pompei P, Ales KL (1987). A new method of classifying prognostic comorbidity in longitudinal studies: development and validation. J Chronic Dis.

[18] Zhang D, Tang X, Shen P (2019). Multimorbidity of cardiometabolic diseases: prevalence and risk for mortality from one million Chinese adults in a longitudinal cohort study. BMJ Open.

[19] de Waard AM, Hollander M, Korevaar JC (2019). Selective prevention of cardiometabolic diseases: activities and attitudes of general practitioners across Europe. Eur J Public Health.

[20] Baygi F, Herttua K, Jensen OC (2020). Global prevalence of cardiometabolic risk factors in the military population: a systematic review and meta-analysis. BMC Endocr Disord.

[21] Zullig LL, Sung AD, Khouri MG (2022). Cardiometabolic comorbidities in cancer survivors: JACC: CardioOncology state-of-the-art review. JACC CardioOncol.

[22] Institute for Public Health (IPH), National Institutes of Health, and Ministry of Health Malaysia (2019). National Health and Morbidity Survey (NHMS) 2019: Vol. I: NCDs – Non-Communicable Diseases: Risk Factors and Other Health Problems.

[23] Tan AK, Dunn RA, Yen ST (2011). Ethnic disparities in metabolic syndrome in Malaysia: an analysis by risk factors. Metab Syndr Relat Disord.

[24] Mahadevan L, Yesudas A, Sajesh PK (2014). Prevalence of genetic variants associated with cardiovascular disease risk and drug response in the Southern Indian population of Kerala. Indian J of Hum Genet.

[25] Bardia A, Olson JE, Vachon CM (2011). Effect of aspirin and other NSAIDs on postmenopausal breast cancer incidence by hormone receptor status: results from a prospective cohort study. Breast Cancer Res Treat.

[26] Sassano A, Platanias LC (2008). Statins in tumor suppression. Cancer Lett.

[27] Takkouche B, Regueira-Méndez C, Etminan M (2008). Breast cancer and use of nonsteroidal anti-inflammatory drugs: a meta-analysis. J Natl Cancer Inst.

[28] Michalaki V, Koutroulis G, Syrigos K (2005). Evaluation of serum lipids and high-density lipoprotein subfrac-tions (HDL2, HDL3) in postmenopausal patients with breastcancer. Mol Cell Biochem.

[29] Saba AB, Ajibade T (2012). Role of Lipoproteins in Carcinogenesis and in Chemoprevention.

[30] Sarfati D (2012). Review of methods used to measure comorbidity in cancer populations: no gold standard exists. J Clin Epidemiol.

[31] Powers BW, Modarai F, Palakodeti S (2020). Impact of complex care management on spending and utilization for high-need, high-cost Medicaid patients. Am J Manag Care.

[32] Hershey DS, Given BA (2020). Improving care coordination for comorbidity and Cancer: a necessity for patients with cancer. Cancer Nurs.

